# MerTK, a risk factor for NASH fibrosis

**DOI:** 10.18632/aging.104210

**Published:** 2020-10-27

**Authors:** Xiaobo Wang, Bishuang Cai

**Affiliations:** 1 Department of Medicine, Columbia University Irving Medical Center, New York, NY 10032, USA; 2 Department of Medicine, Icahn School of Medicine at Mount Sinai, New York, NY 10029, USA

**Keywords:** NASH, GWAS, MerTK, ADAM17, liver fibrosis

Although nonalcoholic fatty liver disease (NAFLD) is more prevalent in older individuals, the underlying mechanisms by which aging processes accelerate NAFLD are not fully understood. NAFLD can progress to nonalcoholic steatohepatitis (NASH), which in turn can lead to the development of cirrhotic liver disease and hepatocellular carcinoma (HCC). Since NAFLD, NASH, and HCC are rapidly increasing in the aging population, understanding the mechanism of how NAFLD progresses to NASH is crucial. NASH is emerging as the leading cause of chronic liver disease and the most rapidly growing indication for liver transplantation worldwide, with liver fibrosis being the most important predictor of liver failure in NASH [1]. However, due to the major gaps in our understanding of the mechanisms of NASH progression, particularly fibrosis, there are currently no FDA-approved medications to treat NASH.

**Figure 1:**
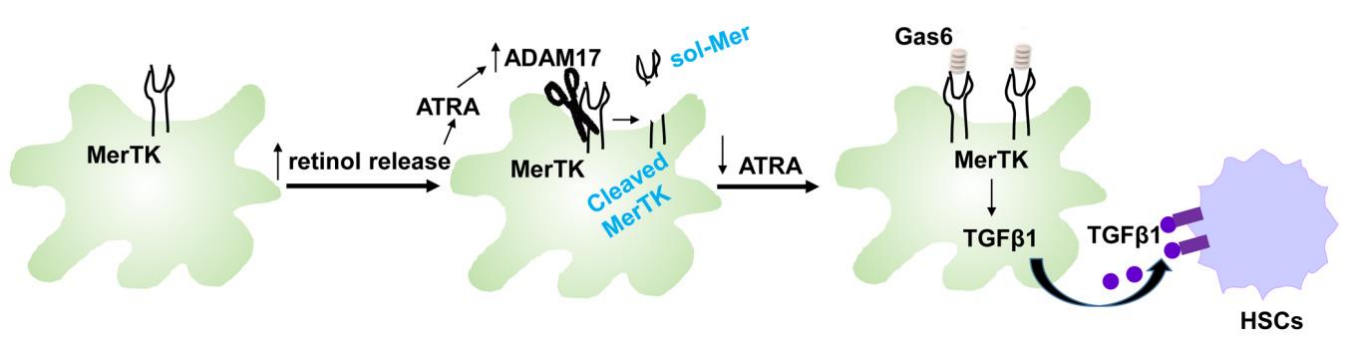
**Summary scheme of MerTK-mediated macrophage HSC crosstalk pathway in fibrotic NASH.** In healthy liver, MerTK in resident macrophages is inactivated due to the absence of GAS6. In steatosis, ATRA-induced MerTK cleavage suppresses MerTK signaling. However, during progression to fibrotic NASH, MerTK signaling is restored by suppression of MerTK cleavage, leading to TGFB1 induction and HSC activation.

Liver fibrosis is driven by the activation of hepatic stellate cells (HSCs) that produce collagen and other types of extracellular matrix. It has been widely reported that liver macrophages, including resident Kupffer cells and infiltrated monocyte-derived macrophages, can activate HSCs through releasing growth factors including TGFβ and PDGF [2]. However, more studies are required to better understand the crosstalk between macrophages and HSCs during NASH progression. Recent GWAS identified MerTK, a tyrosine kinase receptor highly expressed in macrophages, as a potential contributor to liver fibrosis in NASH and hepatitis C (HCV) patients [3,4], but the mechanism linking macrophage MerTK to HSC activation and liver fibrosis is not clear. Cai [5] reported that whole-body and myeloid-specific deletion of MerTK reduced liver fibrosis in a diet-induced NASH model. Mechanistically, MerTK activation by its ligand GAS6 in Kupffer cells induced the production of TGFβ1, leading to hepatic stellate cell (HSC) activation, thereby promoting the progression of NASH fibrosis from steatosis. Moreover, using the cleavage-resistant (*Mertk^CR^*) mouse model, the authors further showed that all-trans retinoic acid (ATRA)–induced ADAM metallopeptidase domain 17 (ADAM17)–mediated MerTK cleavage decreased the levels of MerTK receptor on macrophages and suppressed NASH fibrosis. ATRA is a major active metabolite of retinol stored in quiescent HSCs in healthy liver. In damaged liver, as HSCs are activated, they release retinol. Livers of subjects with NASH show lower levels of retinol than livers of subjects with simple steatosis [6]. Consistent with this concept, Cai et al. found that liver ATRA was decreased in fibrotic livers versus livers with steatosis, leading to an impairment of ATRA-induced MerTK cleavage (Figure 1). The authors showed that increased GAS6 levels during NASH progression may be crucial in the MerTK activation mechanism. This study provides a plausible mechanism underlying MERTK as a genetic risk factor for NASH fibrosis and indicates potential therapeutic strategies to suppress or slow down the progression of NASH fibrosis. Indeed, the authors demonstrated that RU-301, a compound blocking the activation of MerTK by its ligands, and ATRA, which induces MerTK cleavage and reduced MerTK levels on the cell surface of macrophages, decrease liver fibrosis during steatosis-to-NASH progression *in vivo*. However, given that MerTK is critical for maintaining tissue homeostasis in other settings, including atherosclerosis and myocardial infarction [7,8], global MerTK targeting may have undesirable effects. Therefore, a strategy of blocking the MerTK pathway specifically in liver macrophages, for example, via nanotechnology, would be optimal.

In summary, Cai et al. uncover a previously unrecognized function of macrophage MerTK in NASH fibrosis and provide constructive insight on the potential therapeutic targeting of MerTK. However, this study opens up new questions worth exploring. Since MerTK is often seen as a protective regulator for tissue homeostasis, why doesn’t MerTK signaling trigger beneficial effects, including efferocytosis, dampening of inflammation, and resolution of inflammation in the context of NASH? Moreover, some studies have shown that Kupffer cells are protective in non-NASH liver injury models. However, Kupffer MerTK is detrimental in NASH. Does MerTK express in a unique subset of Kupffer cells that promote NASH progression? Finally, what are the mechanisms of decreased liver ATRA and increased GAS6 during NASH?
